# Integrated metabolome and transcriptome analyses of anthocyanin biosynthesis reveal key candidate genes involved in colour variation of *Scutellaria baicalensis* flowers

**DOI:** 10.1186/s12870-023-04591-3

**Published:** 2023-12-15

**Authors:** Fengdan Guo, Renwei Guan, Xinru Sun, Cuicui Zhang, Chenggang Shan, Mengyu Liu, Ning Cui, Ping Wang, Huibin Lin

**Affiliations:** 1 Institute of Chinese Medicine Resources, Shandong Academy of Chinese Medicine, No.7, Yanzishan West Road, Jinan, 250014 PR China; 2 Institute of Industrial Crops, Shandong Academy of Agricultural Sciences, Jinan, 250100 PR China; 3 College of Pharmacy, Shandong University of Traditional Chinese Medicine, Jinan, 250355 PR China

**Keywords:** *Scutellaria baicalensis*, Flower colour, Anthocyanin, Structural genes, Transcription factors

## Abstract

**Background:**

Bright flower colour assists plants attract insects to complete pollination and provides distinct ornamental values. In some medicinal plants, diverse flower colour variations usually imply differences in active ingredients. Compared to the common bluish purple of *Scutellaria baicalensis* flower (SB), the natural variants present rose red (SR) and white (SW) flowers were screened out under the same growing conditions in the genuine producing area Shandong Province, China. However, the mechanism of flower colour variation in *S. baicalensis* was remain unclear. In the present study, we conducted integrated transcriptome and metabolome analyses to uncover the metabolic difference and regulation mechanism in three *S. baicalensis* flowers.

**Results:**

The results showed that 9 anthocyanins were identified. Among which, 4 delphinidin-based anthocyanins were only detected in SB, 4 cyanidin-based anthocyanins (without cyanidin-3-O-glucoside) mainly accumulated in SR, and no anthocyanin but high level of flavanone, naringenin, was detected in SW. The gene expression profile indicated that the key structural genes in the flavonoid and anthocyanin biosynthesis pathway differentially expressed in flowers with different colours. Compared to SB, the down-regulated expression of *F3’5’H*, *ANS*, and *3GT* gene in SR might influence the anthocyanin composition. Especially the InDel site with deletion of 7 nucleotides (AATAGAG) in *F3’5’H* in SR might be the determinant for lack of delphinidin-based anthocyanins in rose red flowers. In SW, the lower expression levels of *DFR* and two *F3H* genes might reduce the anthocyanin accumulation. Notably the SNP site of G > A mutation in the splicing site of *DFR* in SW might block anthocyanin biosynthesis from flavanones and thus cause white flowers. In addition, several key transcription factors, including MYB, bHLH, and NAC, which highly correlated with structural gene expression and anthocyanin contents were also identified.

**Conclusions:**

These results provide clues to uncover the molecular regulatory mechanism of flower colour variation in *S. baicalensis* and promote novel insights into understanding the anthocyanin biosynthesis and regulation.

**Supplementary Information:**

The online version contains supplementary material available at 10.1186/s12870-023-04591-3.

## Background

*Scutellaria baicalensis* Georgi is a perennial herb widely distributed in Asia for its medicinal properties [1]. In China, it was firstly recorded in Shennong Bencao Jing over 2,000 years ago [2]. Recent studies have proved the efficacy of *S. baicalensis* in treating diseases of respiratory system, digestive system, nervous system, and cancer for its rich bioactive components represented by flavonoids, diterpenes, and polysaccharides [3–5]. During the long-term cultivation, *S. baicalensis* produced many variations, including flower colour, leaf size, leaf epidermal hair and so on.

Flower colour is an important character of angiosperms and flower colour variation is the typical representative of plant biodiversity. Studies have shown that flower colour mainly depends on the composition and content of anthocyanins, which play important physiological roles in plants, such as protecting plants against UV radiation and pathogen infection, attracting pollinators, regulating the growth and development under biotic and abiotic stresses [6–8]. In addition, anthocyanins have a variety of biological functions on human health and have shown to be beneficial in preventing and reducing risk of cardiovascular disease, type 2 diabetes, obesity and allergy [9, 10]. Anthocyanins belong to flavonoids and are synthesized through a specific branch of the flavonoid biosynthesis pathway. Firstly, phenylalanine is sequentially catalyzed by phenylalanine ammonia-lyase (PAL), cinnamic acid 4-hydroxylase (C4H) and 4-coumarate-CoA ligase (4CL) to form p-coumaroyl-CoA, which is the common prophase pathway of flavonoid biosynthesis. Then, one p-coumaroyl-CoA with three malonyl-CoA molecules are transformed to colourless dihydrokaempferol (DHK) by chalcone synthase (CHS), chalcone isomerase (CHI), and flavanone 3-hydroxylase (F3H). Catalyzed by dihydroflavonol 4-reductase (DFR), anthocyanidin synthase (ANS), and UDP-glucose flavonoid glycosyltransferases (UFGT), DHKs are converted to orange coloured pelargonidin. Meanwhile, some DHKs are hydroxylated at the 3’ or 3’,5’ positions of the B-ring by flavonoid 3’-hydroxylase (F3’H) or flavonoid 3’,5’-hydroxylase (F3’5’H) to generate precursors of cyanidin and delphinidin, with red and blue colour, respectively [11]. Among which, DFR is one of the key rate-limiting enzymes that control anthocyanin biosynthesis, and can direct flavonoid pathway towards anthocyanin synthesis [12]. *F3’5’H* gene is referred to as “Blue Gene” and has been applied to generate blue colour in some ornamental flowers by transgenic technology [13].

Anthocyanin accumulation is a complex process and is regulated at different levels through multiple genes [14, 15]. At the transcriptional level, MYB, basic helix-loop-helix (bHLH), and WD40 repeat protein form an activation complex to regulate the expression of anthocyanin structural genes [16–19]. Some MYBs act as repressors in anthocyanin biosynthesis, such as *PhMYB27* in petunia, and *MdMYB16* in apple [20–22]. Overexpression of *MdMYB16* in red-fleshed apple callus inhibited the expression of *MdUFGT* and *MdANS*, and eventually inhibited the anthocyanin synthesis [22]. In addition, WRKY, NAC, and some other transcription factors also positively or negatively regulate anthocyanin biosynthesis by affecting the expression levels of structural genes and *MYB* genes. *PbWRKY75* in pear promotes the expression of *PbMYB10b* and anthocyanin late biosynthetic genes (*PbDFR* and *PbUFGT*) to induce anthocyanin synthesis [23]; while, *AtWRKY41* acts as a repressor of anthocyanin biosynthesis through *AtMYB75*, *AtMYB111*, and *AtMYBD* [24]. In apple, overexpression of *MdNAC42* increases the expression of *MdCHS*, *MdCHI*, *MdF3H*, *MdDFR*, *MdANS* and *MdUFGT* by interacting with *MdMYB10* [25]. Recently, epigenetic and post-translational modifications also have been proved to play critical roles in regulating anthocyanin accumulation [26].

The *S. baicalensis* flowers commonly present bluish purple. In our previous studies, we found the natural variants with rose red and white flowers under the same growing conditions in Laiwu Ziguang ecological garden, Shandong Province, China. It is interesting to figure out the cause of these flower colour variation. Multiple omics techniques, especially the combination of metabolome and transcriptome is a useful strategy in identifying the regulation mechanisms of secondary metabolism in a variety of traditional Chinese medicine [27–31]. The available of the whole genome sequence of *S. baicalensis* promotes molecular biology research and provides possibility for better understanding of the regulation of anthocyanin and other active ingredients [32, 33]. In the present study, the flavonoids and phenolic acids among three *S. baicalensis* flowers were characterized and compared using UPLC-MS/MS. Transcriptome analysis was used to identify differential expressed genes which may provide valuable information to understand the mechanism of colour variation. The identified key structural genes and closely correlated transcription factors help to construct a preliminary regulatory network of anthocyanin biosynthesis in *S. baicalensis*. These results provide valuable information to reveal the mechanism of flower colour variation in *S. baicalensis*, and promote novel insights into understanding the anthocyanin biosynthesis and regulation.

## Results

### Anthocyanin and total flavonoid content in *S. baicalensis* flowers with different colours

**Fig. 1:**
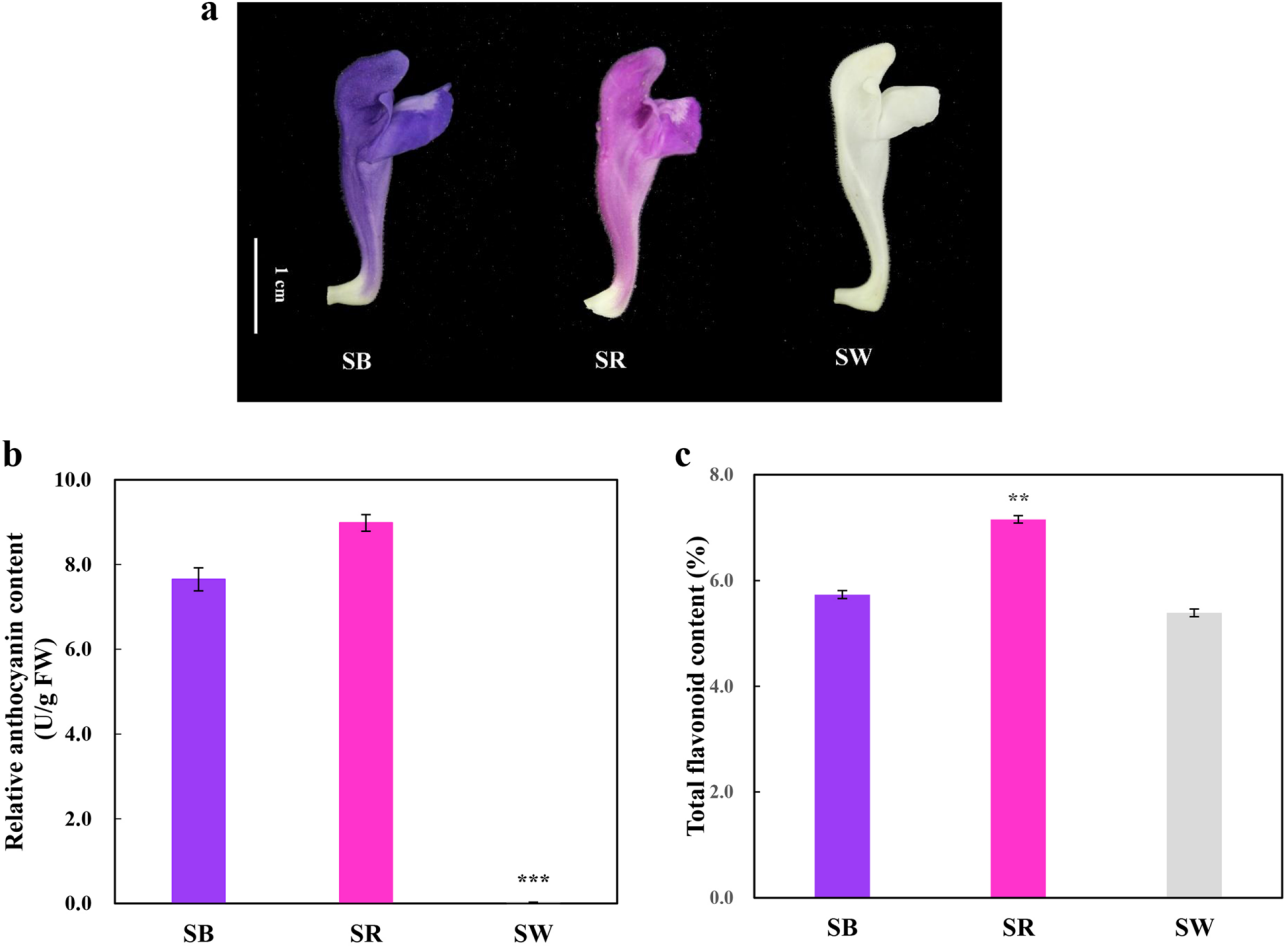
*S. baicalensis* flowers and their anthocyanin and flavonoid content. **a**. Three kinds of *S. baicalensis* flowers with different colours; **b**. Relative anthocyanin content in three kinds of *S. baicalensis* flowers; c. Total flavonoid content in three kinds of *S. baicalensis* flowers

Anthocyanin content is the dominant cause of flower colour. Anthocyanin and many of the active ingredients belong to the flavonoids. The contents of anthocyanin and total flavonoids in *S. baicalensis* flowers of bluish purple (SB), rose red (SR) and white (SW) colour were measured and compared. The relative anthocyanin content of SB, SR and SW was 7.65 U/g FW, 8.98 U/g FW and 0.02 U/g FW, respectively. The anthocyanin content of SW was significantly lower than that of SB and SR. The total flavonoid content of SR was the highest (7.16%), which was significantly greater than that of SB (5.73%) and SW (5.39%) (Fig. 1).

### Metabolite difference in *S. baicalensis* flowers with different colours

**Fig. 2:**
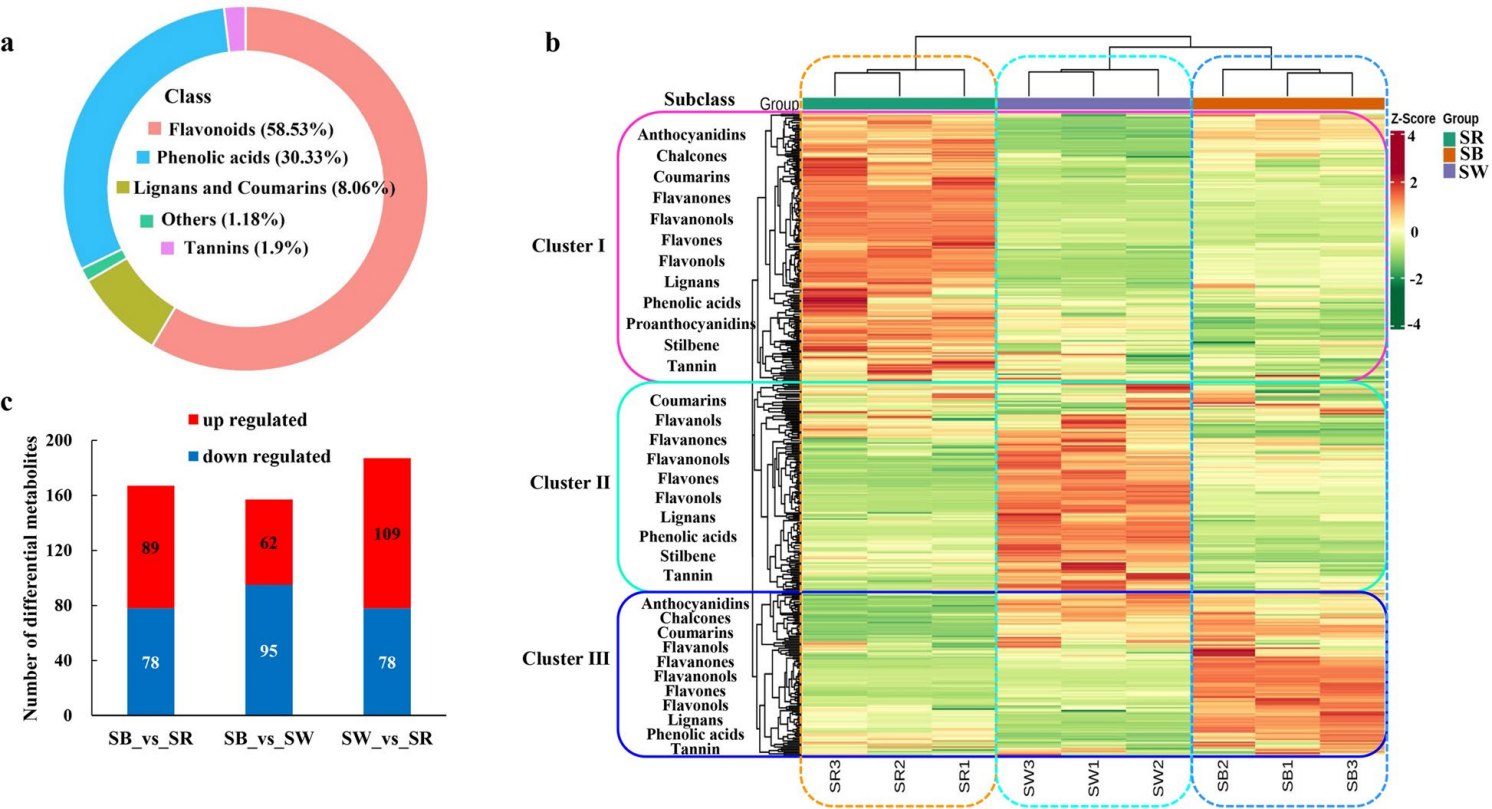
Metabolome profiles of three different flowers of *S. baicalensis*. a. Classification of all identified metabolites. b. HCA of all identified metabolites. Horizontal refers to sample name, and vertical refers to metabolite information. The subclass names of metabolites are labeled on the left of HCA. The color indicates the standardized relative contents of metabolites using unit variance scaling by row. Red represents high content, and green represents low content. c. Numbers of differentially accumulated metabolites among different colours of flowers

To understand the differences in metabolic components that lead to different flower colours in *S. baicalensis*, the flavonoid and phenolic acid compounds, which might cause colour variation, were measured and analyzed. A total of 422 metabolites were identified from three different flowers of *S. baicalensis* (Additional file 1: Table S1). Among which, flavonoid compounds occupied 58.53% (Fig. 2a), including 143 flavones, 57 flavonols, 25 flavanones, 9 anthocyanidins, 8 flavanonols, 3 chalcones, and 2 flavanols. Principal component analysis (PCA) and correlation analysis among samples showed high intra-group repeatability and good inter-group discriminability (Additional file 2: Figure S1). Through hierarchical cluster analysis (HCA) based on the relative contents, metabolites were classified into three clusters showing different accumulation patterns in three different flowers (Fig. 2b). Specifically, metabolites in cluster I prominently accumulated in SR, which represented by cyanidin-3,5-O-diglucoside, dihydroquercetin, apigenin, baicalein-7-O-glucoside, norwogonin, gentisic acid, etc. Metabolites in cluster III mainly distributed in SB, which were primarily composed of delphinidin-3,5-di-O-glucoside, dihydromyricetin-3-O-glucoside, baicalein, baicalin, chrysin, dihydrochrysin, etc. Interestingly, metabolites in cluster II with the highest levels in SW, had no anthocyanin components but flavanones, like naringenin, eriodictyol, carthamidin, and flavones as well as flavonols, such as luteolin, wogonoside, apigenin-5-O-glucoside, myricetin-3-O-glucoside, quercetin-3-O-sophoroside. These results showed that there were certain similarities and, more importantly, specificities in the classification of metabolites in three clusters. Especially anthocyanins, differentially enriched in SB, SR and SW.

According to screening criteria, we identified 253 differentially accumulated metabolites (DAMs) among three colours of flowers (Additional file 1: Table S1). There were 167, 157, and 187 DAMs in SB vs. SR, SB vs. SW, and SW vs. SR, respectively (Fig. 2c). Analysis of global changes of KEGG metabolic pathway showed that anthocyanin, secondary metabolites, flavonoid, flavone and flavonol biosynthesis were the significantly enriched pathways. On the basis of differential abundance score (DA Score), secondary metabolites, flavonoid, flavone and flavonol biosynthesis had no significant change trend in three comparisons. Notably, the anthocyanin biosynthesis pathway obviously tended to be down-regulated in SB vs. SW, up-regulated in SW vs. SR, but presented an insignificant change trend in SB vs. SR (Additional file 3: Figure S2).

**Fig. 3:**
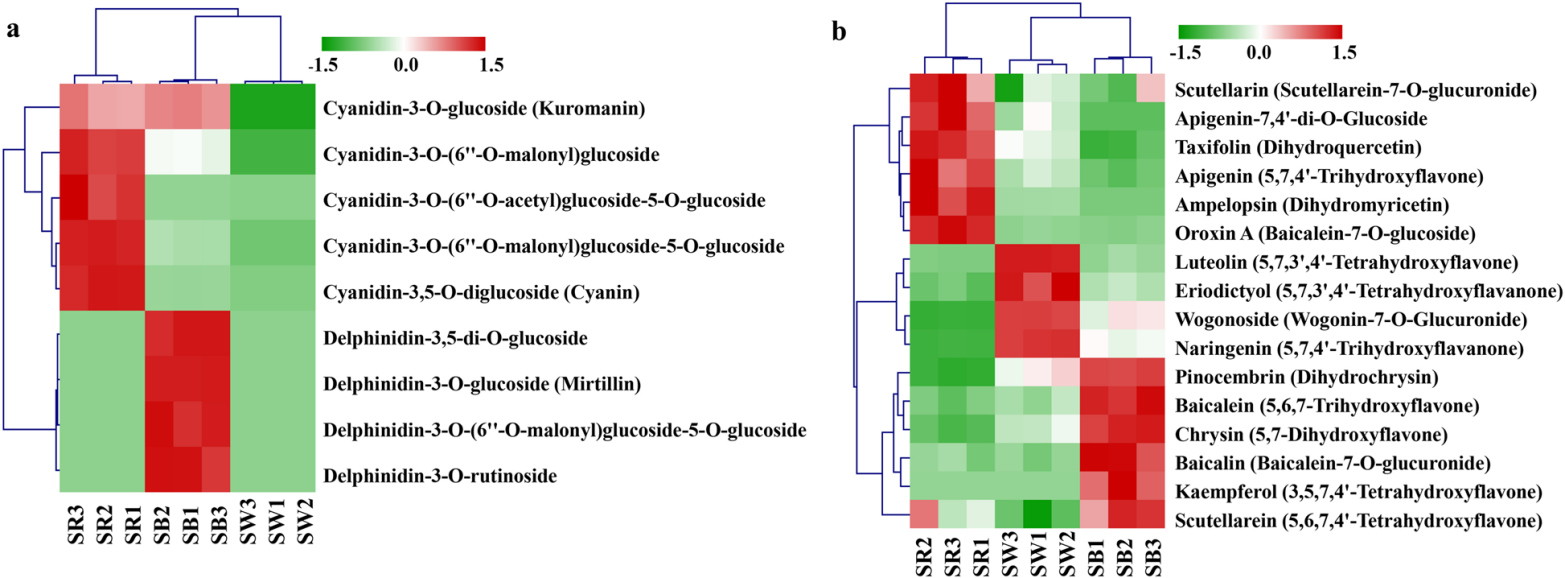
Differentially accumulated patterns of anthocyanins (**a**) and some flavonoids (**b**) among different colours of flowers. Horizontal is the sample name, and vertical is the different anthocyanin compounds. The color indicates the standardized anthocyanin relative contents

In detail, 9 anthocyanins were identified and all of them were differentially accumulated in three different flowers of *S. baicalensis* (Fig. 3a, Additional file 1: Table S1). Five cyanidin-based anthocyanins, including cyanidin-3,5-O-diglucoside, cyanidin-3-O-(6’’-O-malonyl) glucoside, cyanidin-3-O-(6’’-O-acetyl) glucoside-5-O-glucoside, cyanidin-3-O-(6’’-O-malonyl) glucoside-5-O-glucoside, and cyanidin-3-O-glucoside, were highly accumulated in SR, which were averagely 52 and 8,799,089 times as much as those in SB and SW. Four delphinidin-based anthocyanins, including delphinidin-3-O-glucoside, delphinidin-3,5-di-O-glucoside, delphinidin-3-O-rutinoside, and delphinidin-3-O-(6’’-O-malonyl) glucoside-5-O-glucoside, were mainly expressed in SB, which were 13,869,031 times the contents of those in SR and SW. It was speculated that the differential accumulation of red cyanidin and blue delphinidin derivatives led to differential flower colours in SB and SR. Particularly, in SB, except for delphinidin derivatives, there was one cyanidin (cyanidin-3-O-glucoside) with about the same amount as that in SR. This might be why SB appear bluish purple rather than pure blue. In SW, both cyanidin and delphinidin were not detected.

In addition, some other flavonoids with specific accumulation patterns were identified (Fig. 3b). In SR, flavanonols including dihydroquercetin and dihydromyricetin, and some active ingredients, such as apigenin, scutellarin, and oroxin A were all highly accumulated. The baicalein metabolic pathway including pinocembrin, chrysin, baicalein, and baicalin, together with scutellarein were primarily detected in SB. In SW, flavanones containing naringenin, eriodictyol, and active ingredients luteolin, and wogonoside were specially accumulated.

### Transcriptome sequencing and differential gene expression analysis of *S. baicalensis* flowers with different colours

To explore the internal mechanism of anthocyanin and flavonoid differential accumulation, we conducted RNA-seq for three colours of *S. baicalensis* flowers. In total, 424.24 million clean reads with an average of 47.14 million per sample were generated. The Q30 ratio of each sample was 93.50%~94.05%, and the mapping ratio of reads to reference genome was 94.75%~96.03%. Similar to metabolome analysis, PCA and correlation analysis got a clear separation among groups and high correlation in three replicates (Additional file 4: Figure S3). It indicated that the RNA-seq data was of good quality.

**Fig. 4:**
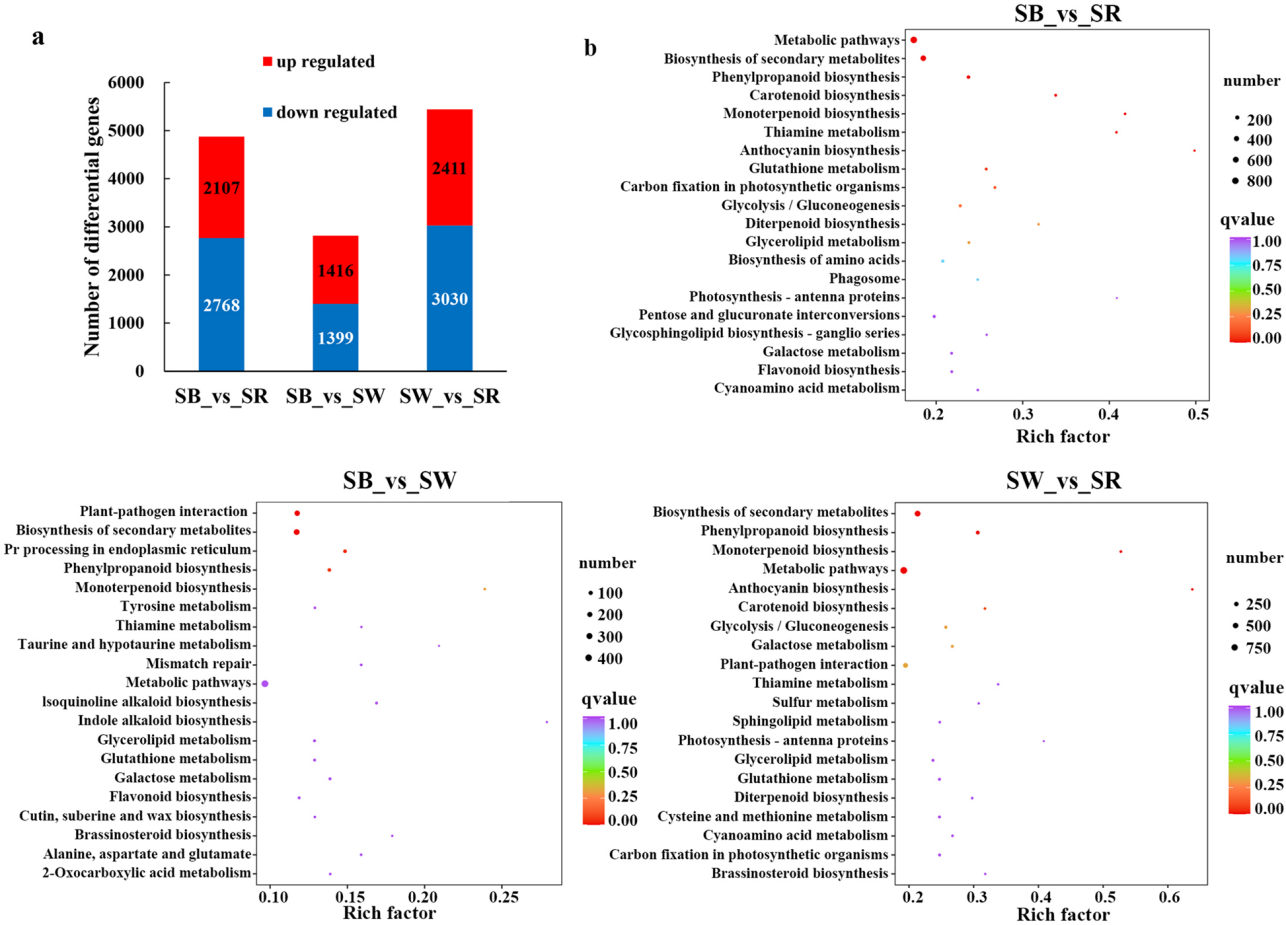
Transcriptome profiles of three different flowers of *S. baicalensis. *a. Numbers of differentially expressed genes of three comparison groups. b. Top 20 enriched KEGG pathways of DEGs of three comparison groups. The x axis represents the Rich factor, and the y axis represents KEGG pathway. The size of the bubbles indicates the number of pathways enriched differential expressed genes. The color represents Q-value of enrichment. The greater the Rich factor, the greater the degree of enrichment. The redder the color, the smaller the Q-value

Through comparing the transcriptome data, 4,875 (2,107 up- and 2,768 down-regulated), 2,815 (1,416 up- and 1,399 down-regulated), and 5,441 (2,411 up- and 3,030 down-regulated) DEGs were identified in SB vs. SR, SB vs. SW, and SW vs. SR, respectively (Fig. 4a, Additional file 5: Table S2). The KEGG enrichment analysis revealed that DEGs in SB vs. SR were mainly enriched in metabolic pathways, biosynthesis of secondary metabolites, phenylpropanoid biosynthesis, carotenoid biosynthesis, anthocyanin biosynthesis, and flavonoid biosynthesis. In SB vs. SW, DEGs were primarily involved in plant-pathogen interaction, biosynthesis of secondary metabolites, phenylpropanoid biosynthesis. Additionally, metabolic pathways and flavonoid biosynthesis were also enriched. In SW vs. SR, biosynthesis of secondary metabolites, phenylpropanoid biosynthesis, metabolic pathways, and anthocyanin biosynthesis were enriched (Fig. 4b).

**Fig. 5:**
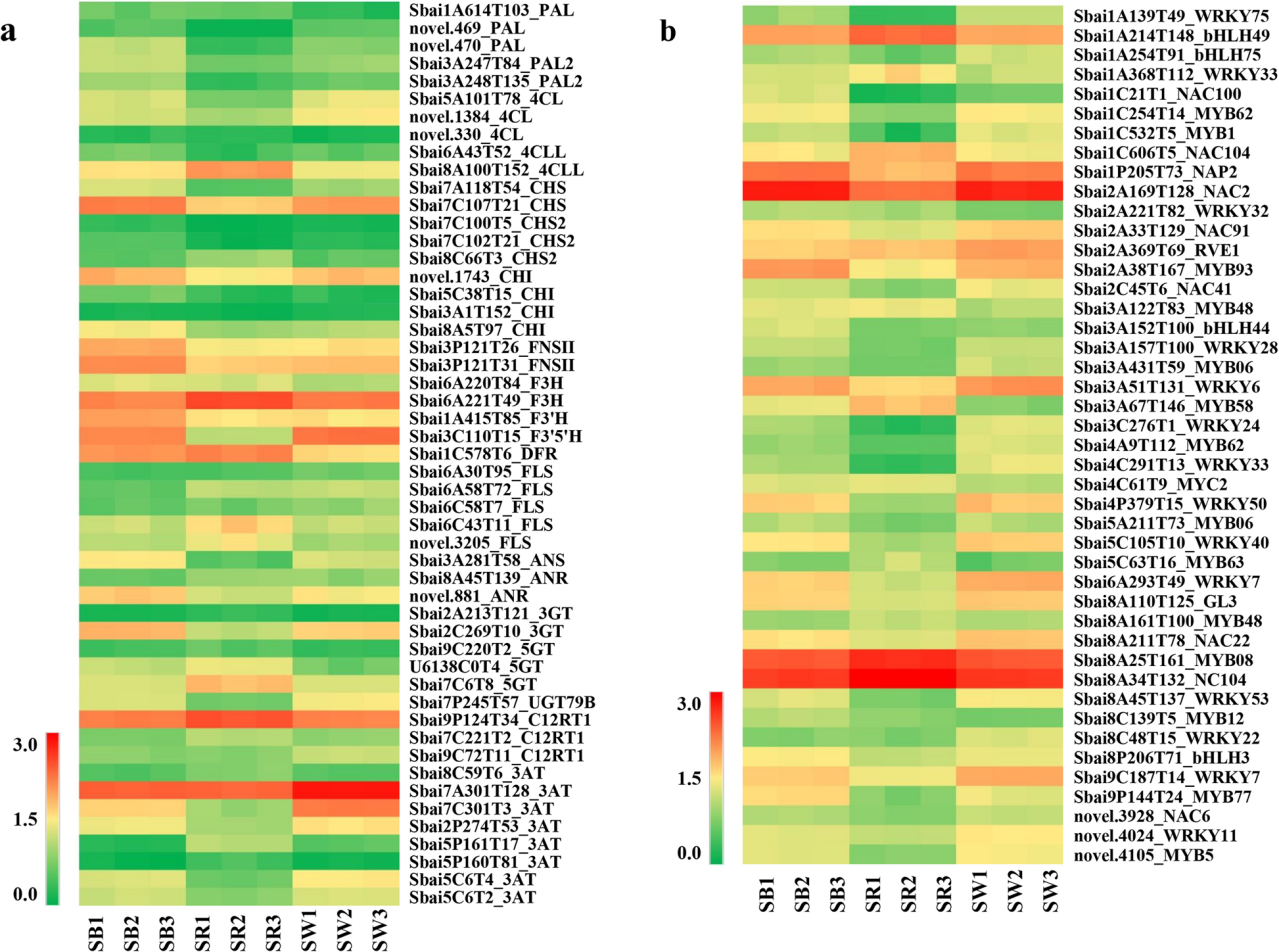
Expression heatmap of 51 DEGs involved in flavonoid and anthocyanin biosynthesis. Cell colours correspond to log_10_ (FPKM + 1): redder cells indicate higher expression, and greener cells indicate lower expression

According to the metabolite difference and KEGG pathway analysis, DEGs involved in flavonoid and anthocyanin biosynthesis pathways (ko00941 and ko00942) were focused in the following analysis. A total of 71 genes were identified in all comparison groups. Through gene annotation analysis, 51 DEGs related to typical flavonoids and anthocyanins were obtained, including five *PAL*, five *4CL*, five *CHS*, four *CHI*, two *FNS II*, two *F3H*, one *F3’H*, one *F3’5’H*, one *DFR*, five *FLS*, one *ANS*, two ANR, nine glycosyltransferase genes, and eight acyltransferase genes (Fig. 5a, Additional file 6: Table S3). Among which, most genes consisted in the upstream of the metabolic pathways generally had low expression levels, like *PAL*, *4CL*, *CHS2*; while genes acting in the downstream that determines the flow of metabolites showed high and differential expression in different groups, such as *F3H*, *F3’H*, *F3’5’H*, *DFR*, and *FNS II*; some anthocyanin modification gene including *3GT* and *5GT* also showed different expression pattern in different groups.

The differentially expressed transcription factors were also analyzed in three colours of *S. baicalensis* flowers. A total of 377 TFs were identified, which belonged to 57 TF families. Of which, AP2/ERF (30/377), WRKY (28/377) and MYB (22/377) were the top three highest families, followed by C2H2 (21/377) and bHLH (19/377) family (Additional file 7: Table S4). MYB, bHLH, WRKY and NAC TF family have been reported regulating gene expression in anthocyanin biosynthesis. After filtering out TFs with the FPKM value lower than 10 in all samples, 44 TFs of above four families were identified and shown with a heatmap (Fig. 5b, Additional file 7: Table S4). Compared to SB, 8 and 23 TFs were up- and down-regulated in SR, while 5 and 5 were up- and down-regulated in SW, respectively. In SR vs. SW group, there were 29 and 10 up- and down-regulated TFs. These TFs might play important roles in the synthesis of anthocyanins.

### Correlation analysis of DEGs with differentially accumulated compounds and TFs associated with anthocyanin synthesis

**Fig. 6:**
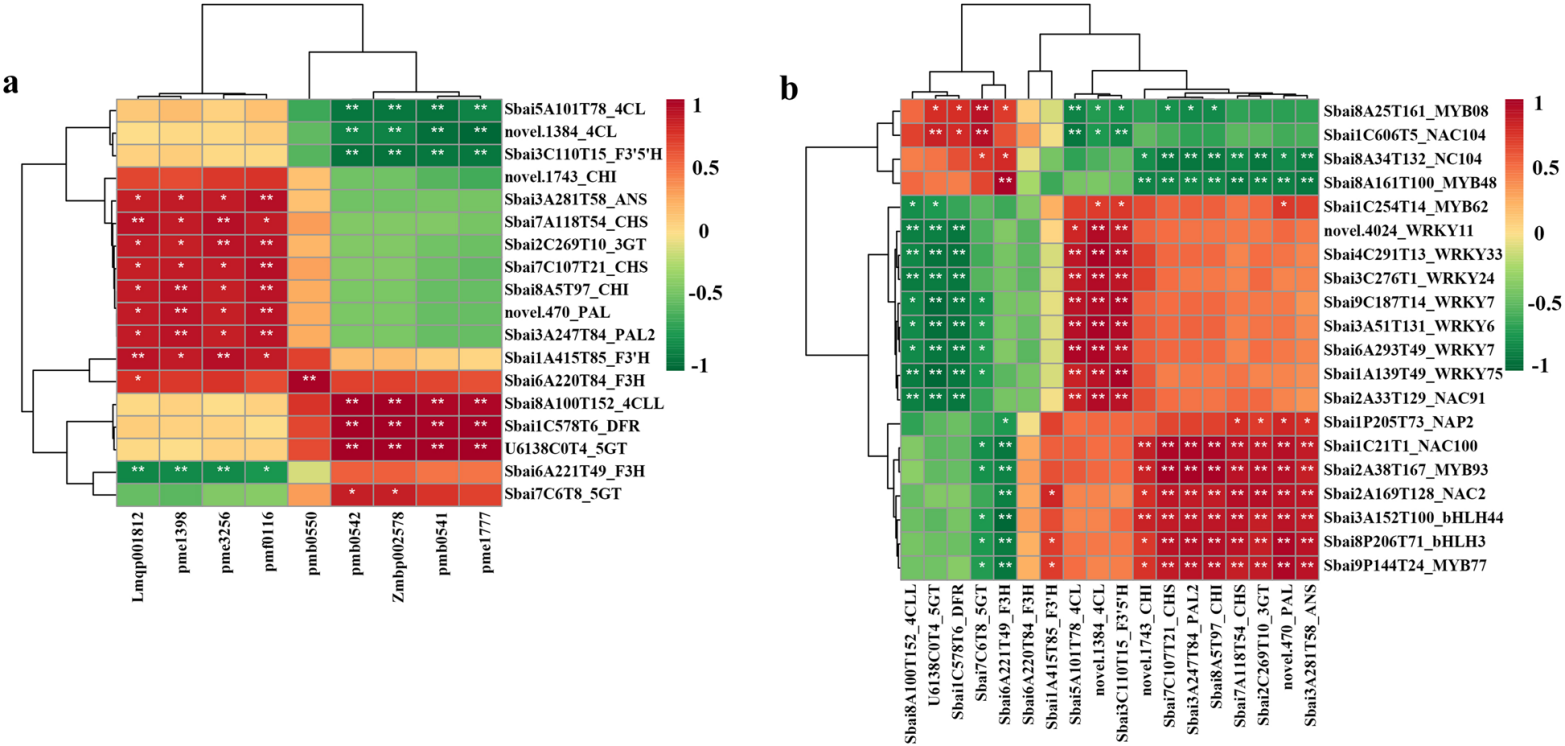
Correlation heatmap between DEGs and differentially accumulated compounds or TFs associated with anthocyanin synthesis. a. Correlation between DEGs and differentially compounds; Lmqp001812: Delphinidin-3-O-(6''-O-malonyl)glucoside-5-O-glucoside; pme1398: Delphinidin-3-O-glucoside; pme3256: Delphinidin-3-O-rutinoside; pmf0116: Delphinidin-3,5-di-O-glucoside; pmb0550: Cyanidin-3-O-glucoside; pmb0542: Cyanidin-3-O-(6''-O-malonyl)glucoside; Zmbp002578: Cyanidin-3-O-(6''-O-acetyl)glucoside-5-O-glucoside; pmb0541: Cyanidin-3-O-(6''-O-malonyl)glucoside-5-O-glucoside; pme1777: Cyanidin-3,5-O-diglucoside. b. Correlation between DEGs and transcription factors. The redder cells represent higher positive correlations, and greener cells indicate higher negative correlations. *. *P* < 0.05; **. *P* < 0.01; ***. *P* < 0.001

To investigate the influencing factors of anthocyanin biosynthesis in *S. baicalensis* flowers, the correlation analysis between DEGs with 9 typical anthocyanin compounds and 44 TFs associated with anthocyanin synthesis were carried out. Through filtering out genes with the FPKM value lower than 10 in all samples, 18 out of 51 structural genes related to anthocyanins were identified (Additional file 6: Table S3). It showed that 9 anthocyanins were significantly correlated with 18 genes (Fig. 6a). Thereinto, cyanidin-based and delphinidin-based anthocyanins gathered together separately and exhibited different correlations with DEGs. Cyanidin-based anthocyanins except for cyanidin-3-O-glucoside were highly positively related to Sbai1C578T6_*DFR*, Sbai7C6T8_*5GT*, and U6138C0T4_*5GT*, while negatively related to two *4CL* genes and Sbai3C110T15_*F3’5’H*. Delphinidin-based anthocyanins together with cyanidin-3-O-glucoside were positively regulated by two *PAL*, two *CHS*, one *CHI*, Sbai6A220T84_*F3H*, Sbai3A281T58_*ANS*, and Sbai2C269T10_*3GT* while negatively related to Sbai6A221T49_*F3H*. It also revealed that 18 structural genes associated with anthocyanin biosynthesis were significantly correlated with 44 transcription factor genes (Fig. 6b, Additional file 7: Table S4). It indicated the potential catalytic and regulatory functions of these genes on anthocyanin biosynthesis.

### Integrated analysis of transcriptome and metabolome of anthocyanin biosynthesis in flowers of *S. baicalensis*

**Fig. 7:**
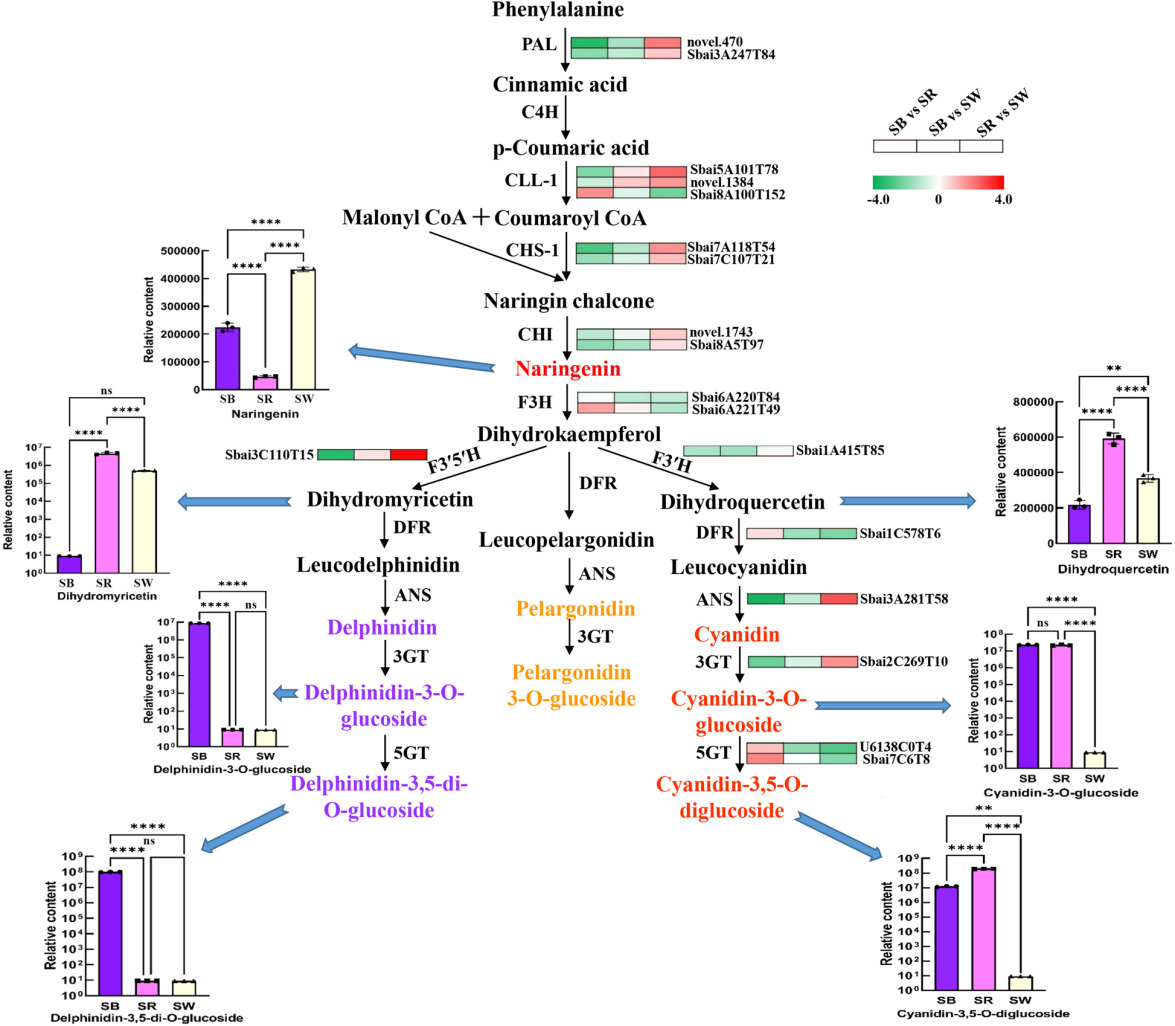
Proposed anthocyanin biosynthesis pathways in *S. baicalensis *with different flower colours**. **The gene expression heatmap was constructed according to log_2_FC (fold change) in three groups (SB vs SR, SB vs SW, and SR vs SW). Column diagrams represented the relative contents of metabolites in three colours of flowers.We have obtained appropriate copyright permission to use and modify the KEGG image depicted in FIGURE & ADDITIONAL FILE

To predict the molecular mechanisms underlying different flower colour of *S. baicalensis*, the transcriptomic and metabolomics data were comprehensively analyzed (Fig. 7). The results showed that delphinidin-3-O-glucoside and delphinidin-3,5-di-O-glucoside, which presented blue, specifically accumulated in flowers of SB but not in SR. F3’5’H is a key enzyme catalyzing anthocyanin synthesis tend to form blue delphinidin pigment [34]. Compared to SB, the expression level of *F3’5’H* gene (Sbai3C110T15) in SR was significantly lower. Besides, the *ANS* (Sbai3A281T58) and *3GT* (Sbai2C269T10) gene were down-regulated in SR than that in SB. Cyanidins, including cyanidin-3-O-glucoside and cyanidin-3,5-O-diglucoside, mainly accumulated in flowers of SR followed by SB. Consistently, two *5GT* genes (U6138C0T4, Sbai7C6T8) had higher expression levels in SR compared to SB. For SW, the content of naringenin, intermediate of anthocyanin synthesis, was significantly higher than that in SB and SR, while the cyanidin and delphinidin were not detected. Compared to SB and SR, the expression levels of two *F3H* genes (Sbai6A220T84, Sbai6A221T49) and one *DFR* gene (Sbai1C578T6) in SW were significantly lower, which might prevent the conversion of naringin to downstream anthocyanin components in SW. It was speculated that these structural genes probably play vital roles in determining the flower colour of *S. baicalensis*.

### Potential regulatory network of anthocyanin biosynthesis in flowers of *S. baicalensis*

**Fig. 8:**
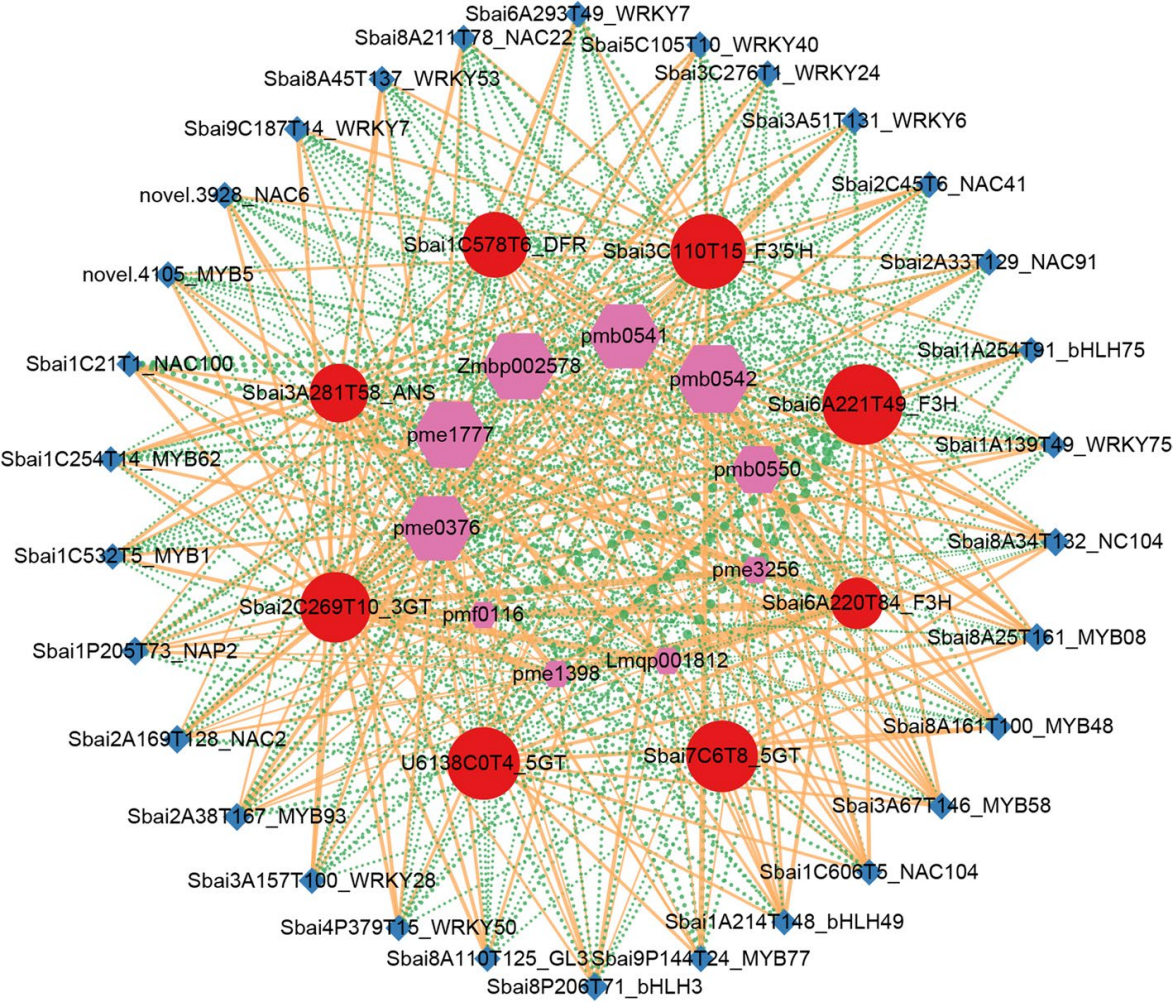
Connection networks among main biosynthetic genes, anthocyanin metabolites and TFs. The red, purple, and blue graphs represent anthocyanin biosynthetic genes, metabolites, and TFs, respectively. The orange and green lines represent positive and negative correlations, respectively. The graph size and line width represent the degree of correlation

To explore the regulatory network of anthocyanin biosynthesis in three colours of flowers of *S. baicalensis*, 8 main biosynthetic genes obtained from integrated transcriptional and metabolic analysis were conducted correlation network analysis with 10 anthocyanin metabolites and transcription factors. As shown in Fig. 8, there were close correlations among key genes, metabolites and TFs. Among which, 40 TFs, including *MYB*, *WRKY*, *NAC* and *bHLH*, showed significantly positive or negative correlations with 8 main structural genes (Additional file 8: Table S5). For instance, the deduced key gene *F3’5’H* (Sbai3C110T15) had highly positive correlations with *NAC91*, *WRKY6*, *GL3* (bHLH), *WRKY53* and *MYB5*, and highly negative correlations with *MYB58*, *bHLH49*, *MYB08* and two *NAC104*. The *DFR* gene was highly positively correlated with *MYC2*, *MYB48*, *MYB58*, *WRKY53* and *MYB5* and negatively correlated with *WRKY11*, *WRKY33*, *WRKY7*, *WRKY24* and *MYB62*. Interestingly, some of the TFs had inverse correlations with *F3’5’H* and *DFR* gene.

According to the transcriptome data [33] of different tissues of *S. baicalensis*, the tissue expression pattern of 8 main structural genes and main TFs were analyzed (Additional file 10: Figure S4). It revealed that 6 structural genes including *F3’5’H*, *DFR*, *5GT* (U6138C0T4), *3GT* and two *F3H* had high expression levels in flower buds or flowers, while *ANS* and *5GT* (Sbai7C6T8) mainly expressed in roots or roots treated by MeJA. Several TFs, such as *MYB08*, *GL3*, two *NAC104*, *MYB06*, *MYB93*, *bHLH44*, *bHLH75*, *bHLH49* and *RVE1*, showed high expression levels in flower buds or flowers.

### Variation analysis of key genes regulating differential flower coloration in *S. baicalensis*

**Fig. 9:**
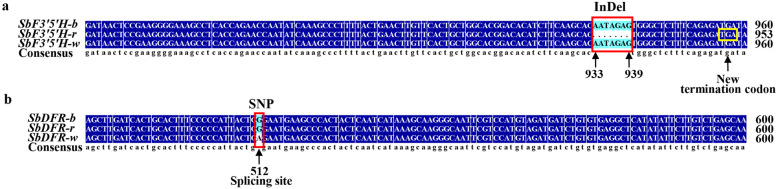
Sequence alignment of *SbF3’5’H* (**a**) and *SbDFR* (**b**) gene from three colours of *S. baicalensis. SbF3’5’H/DFR-b/r/w* represents the *F3’5’H/DFR* gene from SB, SR and SW, respectively. a. The red box shows the 7 bp-deletion of *SbF3’5’H-r* gene from SR, and the yellow box shows the new termination codon caused by frameshift mutation in *SbF3’5’H-r* gene. b. The red box shows the G>A mutation at the splicing site of *SbF3’5’H-w* gene from SW

To figure out the variation of 8 structural genes in three *S. baicalensis*, the sequence comparisons were conducted to identified SNP (single nucleotide polymorphism) and InDel (insertion and deletion) variant sites. A total of 98 SNPs and 19 InDels were identified (Additional file 9: Table S6). Of which, 44 SNPs and 3 InDels were located in the exon region of structural genes, 3 SNPs and 1 InDel were located in the upstream of structural genes. Notably, one InDel site with deletion of 7 nucleotides (AATAGAG) in the exon region of *F3’5’H* gene resulted in frameshift mutation in SR. One SNP of G > A mutation located in the splicing site in *DFR* gene might cause splicing defect in SW (Fig. 9). These results provided clues for us to uncover the regulatory mechanism of flower colour variation in *S. baicalensis*.

### qRT-PCR verification of transcriptomic data

**Fig. 10:**
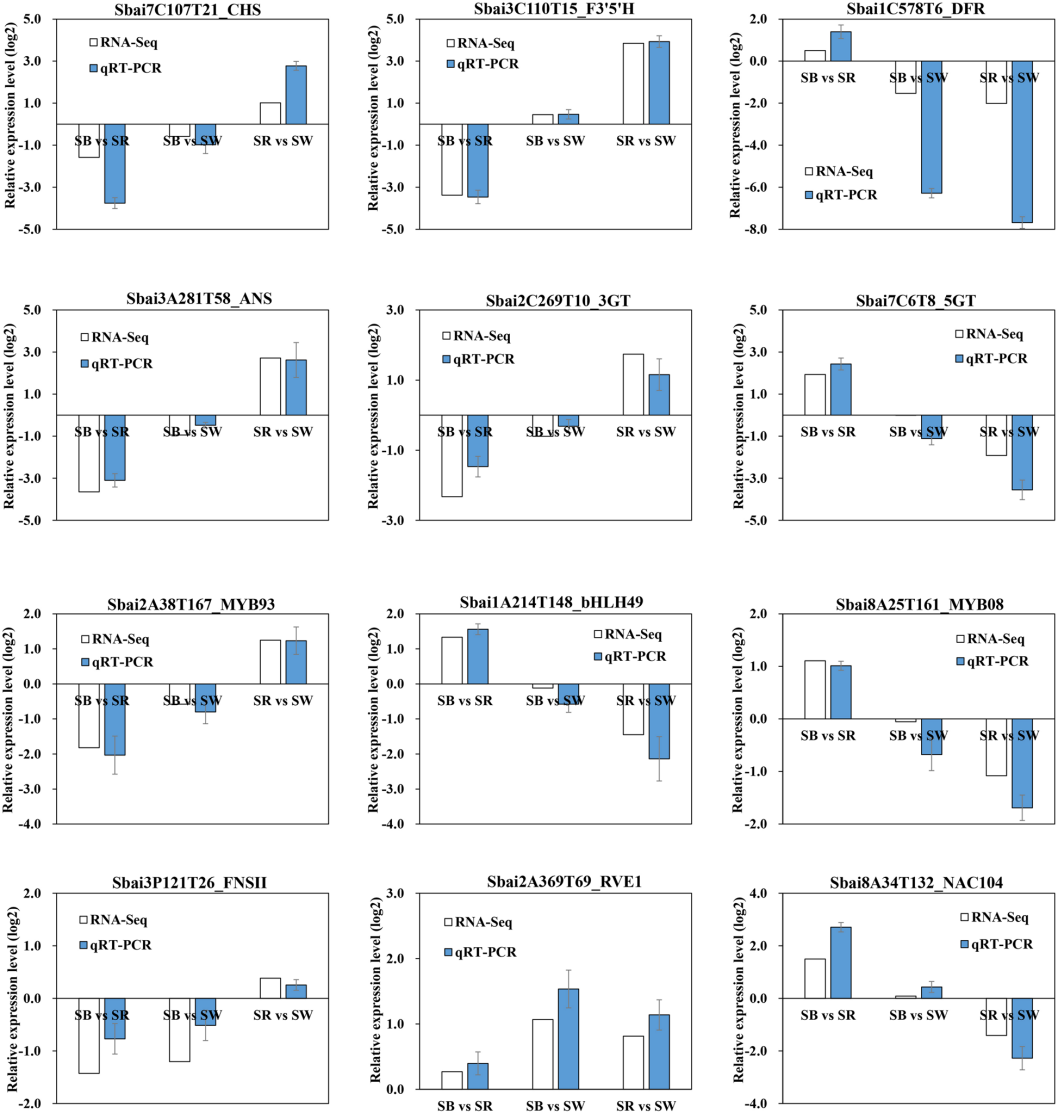
qRT-PCR verification of genes related to anthocyanin biosynthesis*. *White bar charts represent the relative expression levels (log_2_FC) in three groups (SB vs SR, SB vs SW, and SR vs SW) from RNA-seq data. Blue bar charts represent the relative expression levels calculated by 2^–^^ΔΔCt^ from qRT-PCR data. The values of qRT-PCR data are means of three biological replicates, and error bars represent ±SE (*n* = 3)

To evaluate the credibility of the transcriptomic data, 12 genes related to anthocyanin biosynthesis including 7 structural genes and 5 transcription factors were selected to conduct qRT-PCR analysis. The results indicated that the expression patterns of 12 genes in three comparison groups were highly in accordance with the relative expression levels (log_2_ Fold Change) obtained from RNA-seq (Fig. 10).

## Discussion

### Anthocyanin metabolite difference in three colours of *S. baicalensis* flowers

Anthocyanins are well known water-soluble pigments which coloring plant flowers and fruits a red, purple, or blue hue. Recently, the composition and difference of anthocyanin in flowers with diverse colours have been studied. In *Salvia miltiorrhiza*, cyanidin 3,5-O-diglucoside, cyanidin 3-O-galactoside, and malvidin 3,5-diglucoside were considered the main differentially accumulated anthocyanin between purple and white flower [28]. For *Carthamus tinctorius*, the colour difference of white, yellow, light red, and deep red flowers was mainly caused by C-glucosylquinochalcones [30]. In previously reported *S. baicalensis* from Shanxi Province of China, cyanidin 3-rutinoside and delphin chloride tend to be the major anthocyanin in purple-red and purple petals, respectively [35]. In general, traditional Chinese medicine (TCM) has the characteristics of geo-authenticity and highly heterozygous genome. The genetic bases of the same kind of TCM derived from different geographical environments might vary greatly. Shandong, Shanxi and Hebei Province are the three genuine producing area of *S. baicalensis* in China. In the present study, the *S. baicalensis* with bluish purple (SB), rose red (SR) and white (SW) flowers from Shandong Province were taken as the study object to explore the component difference and molecular mechanism of anthocyanin accumulation.

It showed that the relative anthocyanin content of SR was slightly higher than that of SB, and they both significantly higher than that of SW (Fig. 1). SR owned the highest total flavonoid content, and that of SB and SW had little difference. By flavonoid and phenolic acid-targeted metabolome, 422 metabolites were identified. Hierarchical clustering showed that cluster I which prominently accumulated in SR had the most metabolites, followed by SW (cluster II) and SB (cluster III) (Fig. 2b). This result was in line with the total flavonoid content. KEGG enrichment analysis of differentially accumulated metabolites also confirmed that anthocyanin accumulation between SB and SR had minor difference, but showed significant difference with SW (Additional file 3: Figure S2).

Through comparing and analyzing of anthocyanin content, we found that 4 delphinidin-based anthocyanins were only accumulated in SB, 4 cyanidin-based anthocyanins (cyanidin-3-O-glucoside not in) were mainly detected in SR, and no anthocyanin was detected in SW in this study. Different from other anthocyanins, the content of cyanidin-3-O-glucoside in SB and SR had insignificant difference. Thus, we deduced that the colour of SR was attributed to the accumulation of red cyanidin only, and the bluish purple colour of SB rather than pure blue was due to a proper ratio of delphinidin to cyanidin. While in SW, absence of both cyanidin and delphinidin caused the white colour. These results were different from that of *S. baicalensis* from Shanxi Province of China.

Besides, some flavonoid intermediates were differentially accumulated in three colours of flowers. Based on the anthocyanin biosynthetic pathway, high levels of dihydroquercetin, dihydromyricetin and cyanidin-based anthocyanins, low content of delphinidin-based anthocyanins in SR indicated that enzymes converting flavanonols to anthocyanins might have different substrate preference. In SW, deficiency of anthocyanins while high accumulation of the precursor substances flavanones portended a defect in the enzyme catalyzing flavanones to anthocyanins.

### Key genes involved in anthocyanin biosynthesis in flowers of *S. baicalensis*

We conducted transcriptome analysis on *S. baicalensis* flowers with different colours, and obtained 4,875, 2,815, and 5,441 DEGs in three comparison groups, respectively. After KEGG enrichment analysis, the flavonoid and anthocyanin biosynthesis pathways were focused, and structural genes on the pathways and related transcription factors were identified. Through correlation analysis, 18 anthocyanin biosynthesis genes were significantly correlated with 9 anthocyanins and 44 transcription factor genes.

Integrated transcriptome and metabolome analyses showed that the expression levels of *F3’5’H*, *ANS*, and *3GT* gene was significantly down-regulated in SR, which might influence the anthocyanin composition in SR. *F3’5’H* gene is responsible for converting dihydrokaempferol to dihydromyricetin to synthesize delphinidin-based anthocyanins. Several cut flower plants, like rose, carnation, and chrysanthemum, cannot generate blue colour because of lack of normal enzyme activity of flavonoid 3’,5’-hydroxylase [13]. In the present study, the deletion of 7 nucleotides in *F3’5’H* gene in SR which result in frameshift mutation probably give rise to premature transcription termination, and it might be the determinant for lack of delphinidin-based anthocyanins in rose red flowers. In SW, the expression levels of *DFR* gene and two *F3H* genes were significantly lower compared to SB and SR. In the study of natural variations of *DFR* in *Solanum* showed that the splicing site of *DFR* gene was important for maintaining enzyme function, and mutations in splicing site resulted in altered splicing and anthocyanin-free flowers [36]. Our study indicated that the G > A mutation in the splicing site of *DFR* gene in SW might lead to splicing defect and block the biosynthesis of anthocyanins, thus produced white flowers. The biologic activities of mutated *F3’5’H* and *DFR* gene will be further studied.

### Transcription factors correlated with anthocyanin biosynthesis in flowers of *S. baicalensis*

Regulatory network and tissue expression pattern analysis identified several TFs, including *MYB08*, *GL3*, two *NAC104*, *MYB06*, *MYB93*, *bHLH44*, *bHLH75*, *bHLH49*, *RVE1*, that significantly correlated with *F3’5’H* or *DFR* gene and specifically highly expressed in flowers or flower buds. These TFs might participate in the regulation of structural gene expression during accumulation of cyanidin-based and delphinidin-based anthocyanins in flowers of *S. baicalensis*.

## Conclusions

The mechanism underlying colour variation of *S. baicalensis* flowers was analyzed using transcriptome and metabolome profiling. Our results showed that 9 anthocyanin compounds, 18 structural genes and 44 transcription factors related to anthocyanin biosynthesis were identified from three comparison groups. Differential accumulation of delphinidin-based anthocyanins between SB and SR, and lack of anthocyanins in SW were responsible for the flower colour variation of *S. baicalensis*. Integrated analysis of transcriptome and metabolome revealed that the low expression levels of *F3’5’H*, *ANS*, *3GT* in SR and *DFR*, *F3H* in SW might reduce delphinidin-based anthocyanin and total anthocyanin synthesis in SR and SW, respectively. Especially, we identified one InDel site in *F3’5’H* gene from SR and one SNP site in *DFR* gene from SW, which might be the determinants in the formation of rose red and white flowers. Several key TFs, including MYB, bHLH, and NAC, highly correlated with structural gene expression and anthocyanin contents were also identified. The results promote our understanding on the molecular mechanism of colour variation in *S. baicalensis* flowers and provide novel insights into the underlying mechanism of anthocyanin biosynthesis and regulation.

## Methods

### Plant materials

Three-years old *S. baicalensis* with bluish purple (SB), rose red (SR) and white (SW) flowers were grown in Laiwu Ziguang ecological garden, Shandong Province, China. Three kinds of fresh flowers were collected from healthy plants at full-bloom stage in July 2022. Some materials were used directly or dried for component detection, the others were frozen immediately in liquid nitrogen and stored at -80 °C for RNA and metabolite extraction. Three biological replicates were performed in this study.

### Measurement of relative anthocyanin content

For three colours of flowers, 0.2 g of fresh samples were ground with 1 mL methanol containing 0.1% HCl (methanol: concentrated hydrochloric acid = 99:1, v/v), transferred to 10 mL centrifuge tube and washed for another two times. The final volume was diluted to 5 mL with methanol (containing 0.1% HCl). The tissue homogenate was oscillated for 30 s, incubated overnight at 4℃, and oscillated every 8 h. The samples were centrifuged at 4 °C, 12,000 g for 10 min, and the supernatant was used for measuring the absorbance at 530 nm with a microplate spectrophotometer (Bio-Rad xMark). Methanol (containing 0.1% HCl) was used as the blank control. The relative anthocyanin content Q (U/g FW) = A_530_/M, and M represents the weight of the sample.

### Measurement of total flavonoid content

The aluminum nitrate colorimetric method was used to determine the total flavonoid content in *S. baicalensis* flowers referring to Jiang et al. (2020) [28] with slight change. Dried samples were adopted and the results were calculated based on dry weight. Using rutin as the standard substance, the calibration curve equation was gained as Y = 12.881X − 0.0174 with R² = 0.9995.

### Metabolite extraction and detection

Three colours of *S. baicalensis* flowers were vacuum freeze-dried and ground separately to powder by a grinding mill (MM 400, Retsch). Then, 50 mg of powder was dissolved in 1.2 mL 70% methanol aqueous (V/V = 70%). After oscillating for 6 times at 30-min intervals, the mixture was centrifuged at 12,000 rpm for 3 min and filtered through a 0.22-µm microporous membrane for ultra-performance liquid chromatography-tandem mass spectrometry (UPLC-MS/MS) analysis. The sample extracts were analyzed using UPLC (SHIMADZU Nexera X2, https://www.shimadzu.com.cn/) and MS spectrometer (Applied Biosystems 4500 QTRAP, https://www.thermofisher.cn/cn/zh/home/brands/applied-biosystems.html). The UPLC was conducted with the following conditions: Agilent SB-C18 column (1.8 μm, 2.1 mm × 100 mm); mobile phase, phase A was ultrapure water (add 0.1% formic acid) and phase B was acetonitrile (add 0.1% formic acid). Sample measurements were performed with a gradient program that employed the starting conditions of 95% A, 5% B. Within 9 min, a linear gradient to 5% A, 95% B was programmed, and a composition of 5% A, 95% B was kept for 1 min. Subsequently, a composition of 95% A, 5.0% B was adjusted within 1.1 min and kept for 2.9 min. The flow rate was set to 0.35 mL/min, the column temperature was 40 °C, and the injection volume was 4 µL. The MS parameters were as follows: ion source, electrospray ionization (ESI); source temperature, 550 °C; the positive and negative ion spray voltage (IS) were 5,500 V and − 4,500 V, respectively; the ion source gas I (GSI), gas II (GSII), and curtain gas (CUR) were 50, 60 psi, and 25 psi, respectively; the collision-induced ionization parameter was set to high. Triple quadrupole (QQQ) and linear ion hydrazine-flight time (LIT) scans were conducted.

### Metabolic data analysis

According to the MWDB database built by the MetWare Biotechnology Co., Ltd. (Wuhan, China), the qualitative analysis of metabolic data was carried out based on secondary spectral information. The quantification of metabolites was accomplished by multiple reaction monitoring (MRM) mode [37, 38]. The scanned mass spectrum data were analyzed by Analyst 1.6.3 software. For quality assessment of metabolic data, unsupervised principal component analysis (PCA) and hierarchical cluster analysis (HCA) were performed using R software. The data was normalized using unit variance scaling (UV) by row before unsupervised PCA and HCA. The Pearson correlation coefficient, calculated by built-in cor function of R software, was taken as the evaluation index of correlation among biological replicates. Before differential analysis, the PCA and orthogonal partial least squares-discriminant analysis (OPLS-DA) were carried out to observe the variation between and within groups [39]. The DAMs between groups were screened by the criteria of |log_2_ (fold change) | ≥ 1 and variable importance in projection (VIP) ≥ 1. The identified metabolites were annotated and mapped using the Kyoto Encyclopedia of Genes and Genomes (KEGG) database. Subsequently, the DAMs were performed KEGG pathway clustering and enrichment analysis [40].

### RNA sequencing and data analysis

Total RNA extracted from three colours of *S. baicalensis* flowers were conducted quantitative and qualitative analysis using the NanoPhotometer® spectrophotometer (IMPLEN, CA, USA) and Bioanalyzer 2100 system (Agilent Technologies, CA, USA). A total amount of 1 µg RNA per sample was used as for the RNA sample preparations. Sequencing libraries were generated using NEBNext®UltraTM RNA Library Prep Kit for Illumina® (NEB, USA) following manufacturer’s recommendations and index codes were added to attribute sequences to each sample. At last, the libraries were sequenced on an Illumina platform and 150 bp paired-end reads were generated.

Clean reads were obtained through filtering of original data by fastp [41] and mapped to reference genome of *S. baicalensis* (https://ngdc.cncb.ac.cn/gwh/Assembly/10411/show) using HISAT 2 [42]. Through StringTie software, the mapped reads of each sample were assembled in a reference-based approach [43]. Subsequently, quantitative analysis of gene/transcript expression levels were performed and normalized to FPKM (Fragments Per Kilobase of transcript per Million fragments mapped). DESeq2 was used to analyze the differentially expressed genes (DEGs) between two groups with the threshold of |log_2_Fold Change| ≥ 1 and false discovery rate (FDR) < 0.05 [44, 45]. Then, screened DEGs were performed functional annotation, classification and enrichment analysis based on KEGG, GO (Gene Ontology), NR (Non-Redundant Protein Sequence Database), Swiss-Prot (manually annotated and reviewed protein sequences;), TrEMBL (Translation of EMBL), and KOG (Clusters of orthologous groups for eukaryotic complete genomes) database [17–19, 46–48]. In addition, alternative splicing, gene variation, and protein interaction analysis of DEGs were conducted using rMATS [49], GATK [50], Diamond [51] software, respectively.

### Combined analysis of metabolome and transcriptome

The correlation analysis of DEGs and DAMs obtained from transcriptomic and metabolomic profiles were conducted using the cor function in R. The genes and metabolites with absolute correlation coefficient > 0.8 and *p* value < 0.05 were screened out and showed in the nine-quadrant plot, clustering heatmap, and correlation network diagram. The key genes and transcription factors with high linkage value with DAMs of anthocyanin were used to draw the interactive networks using Cytoscape software.

### Analysis of tissue expression pattern

The transcriptome data of different tissues (root, stem, leaf, flower, bud) and root treated with MeJA of *S. baicalensis* were obtained from the SRA database of NCBI website (https://www.ncbi.nlm.nih.gov/sra/) [33]. After quality control with Trimmomatics and Fastp software, the sequencing data were compared to the reference genome (https://ngdc.cncb.ac.cn/gwh/Assembly/10411/show) [32]of *S. baicalensis* using HISAT2 [42]. Then Feature Counts was used to perform quantitative analysis. The tissue expression pattern of anthocyanin biosynthesis genes and related TFs were analyzed referring to this transcriptome data and drew the expression heatmap with TBtools software [52].

### qRT-PCR verification of RNA-seq

Total RNA of *S. baicalensis* flowers was reverse transcribed using the PrimeScript™ RT reagent Kit with gDNA Eraser (TaKaRa, Dalian, China). The quantitative real-time polymerase chain reaction (qRT-PCR) was performed according to the TB Green Premix Ex Taq II Kit (TaKaRa, Tokyo, Japan) on CFX96 Real-Time PCR Detection System (Bio-Rad). Twelve genes were selected for expression analysis with *18 S rRNA* as the internal reference gene. The primers were designed by PerlPrimer and listed in Additional file 11: Table S7. Each gene was conducted three replicates. The relative gene expression levels were calculated using the 2^–ΔΔCt^ method and compared with RNA-seq data.

### Supplementary Information

**Additional file 1: Table S1.** Total metabolites and differential accumulated metabolites in three different flowers of *S. baicalensis.*

**Additional file 2: Figure S1.** Principal component analysis and repeated correlation assessment of metabolome data. a. Principal component analysis among samples. PC1 represents the first principal component, PC2 represents the second principal component, PC3 represents the third principal component, and percentage represents the interpretation rate of this principal component to the data set. Each point in the figure represents a sample, and samples in the same Group are represented by the same color. Different group are distinguished by different colours. b. Correlation analysis among samples. The vertical and diagonal lines represent different sample names, and different colours represent different Pearson correlation coefficients.

**Additional file 3: Figure S2.** The top 20 enriched KEGG pathways of DAMs shown by DA score in SB vs SR (a), SB vs SW (b) and SW vs SR (c). X axis represents the DA score. Y axis represents KEGG pathways. DA Score reflects the overall change of metabolites. A score of 1 indicates an upward trend in the expression of all identified metabolites in this pathway, and a score of -1 indicates a downward trend. The length of the line segment represents the absolute value of the DA Score. The dot size indicates the number of differentiated metabolites in the pathway, and the larger the dot, the more metabolites. The color of the line segment and dot reflects the P-value size. The closer it is to red, the smaller the P-value, and the closer it is to purple, the larger the P-value.

**Additional file 4: Figure S3.** Principal component analysis and correlation analysis of transcriptome data. a. Principal component analysis among samples. b. Correlation analysis among samples.

**Additional file 5: Table S2.** DEGs in three comparison groups of *S. baicalensis* flowers with different colours.

**Additional file 6: Table S3.** DEGs involved in flavonoid and anthocyanin biosynthesis pathway (ko00941 and ko00942).

**Additional file 7: Table S4.** Differentially expressed transcription factors in *S. baicalensis* flowers with different colours.

**Additional file 8: Table S5.** The correlations among key biosynthetic genes, metabolites and TFs in *S. baicalensis* flowers with different colours.

**Additional file 9: Table S6. **SNPs and InDels identified from structural genes in anthocyanin biosynthesis.

**Additional file 10: Figure S4.** Expression patterns of structural genes and main TFs associated with anthocyanin biosynthesis in different tissues of *S. baicalensis*. The gene expression level was expressed by TPM value and normalized by row. The redder cells indicate higher expression, and greener cells indicate lower expression.

**Additional file 11: Table S7. **Specific primers of qRT-PCR analysis.

## Data Availability

The datasets supporting the conclusions of this article are included within the article and its Additional files. The transcriptome data generated from this study were available in the Sequence Read Archive (SRA) of NCBI with the accession number of SRP447599 (https://www.ncbi.nlm.nih.gov/search/all/?term=SRP447599).

## Abbreviations

3GT: Anthocyanidin 3-O-glucosyltransferase
4CL: 4-coumarate-CoA ligase
5GT: Anthocyanidin 3-O-glucoside 5-O-glucosyltransferase
ANS: Anthocyanidin synthase
bHLH: Basic Helix-Loop-Helix
C4H: Cinnamic acid 4-hydroxylase
CHI: Chalcone isomerase
CHS: Chalcone synthase
DAMs: Differentially accumulated metabolites
DEGs: Differentially expressed genes
DFR: Dihydroflavonol 4-reductase
DHK: Dihydrokaempferol
F3’H: Flavonoid 3’-hydroxylase
F35’H: Flavonoid 3’,5’-hydroxylase
F3H: Flavanone 3-hydroxylase
FLS: Flavonol synthase
FNS II: Flavone synthase II
FPKM: Fragments per kilobase per million
GO: Gene Ontology
HCA: Hierarchical cluster analysis
InDel: Insertion and deletion
KEGG: Kyoto Encyclopedia of Genes and Genomes
KOG: Clusters of orthologous groups for eukaryotic complete genomes
MRM: Multiple Reaction Monitoring
MYB: V-Myb Avian Myeloblastosis Viral Oncogene Homolog
NR: Non-Redundant Protein Sequence Database
OPLS-DA: Orthogonal partial least squares-discriminant analysis
PAL: Phenylalanine ammonia-lyase
PCA: Principal component analysis
SB: Bluish purple
SE: Standard error
SNP: Single nucleotide polymorphism
SR: Rose red
SW: White
Swiss-Prot: Manually annotated and reviewed protein sequences
TCM: Traditional Chinese medicine
TFs: Transcription factors
TrEMBL: Translation of EMBL
UFGT: UDP-glucose flavonoid glycosyltransferases
UPLC-MS/MS: Ultra-performance liquid chromatography-tandem mass spectrometry
UV: Unit variance scaling
