# Supplementary material for: Integrated metabolome and transcriptome analyses of anthocyanin biosynthesis reveal key candidate genes involved in colour variation of Scutellaria baicalensis flowers
Source: BMC Plant Biol. 2023 Dec 15;23:643. doi: 10.1186/s12870-023-04591-3 (PMC10722828; doi:10.1186/s12870-023-04591-3)
Supplement: Supplementary file 2 — Additional file 2: Figure S1. Principal component analysis and repeated correlation assessment of metabolome data. a. Principal component analysis among samples. PC1 represents the first principal component, PC2 represents the second principal component, PC3 represents the third principal component, and percentage represents the interpretation rate of this principal component to the data set. Each point in the figure represents a sample, and samples in the same Group are represented by the same color. Different group are distinguished by different colours. b. Correlation analysis among samples. The vertical and diagonal lines represent different sample names, and different colours represent different Pearson correlation coefficients. [file 12870_2023_4591_MOESM2_ESM.docx]

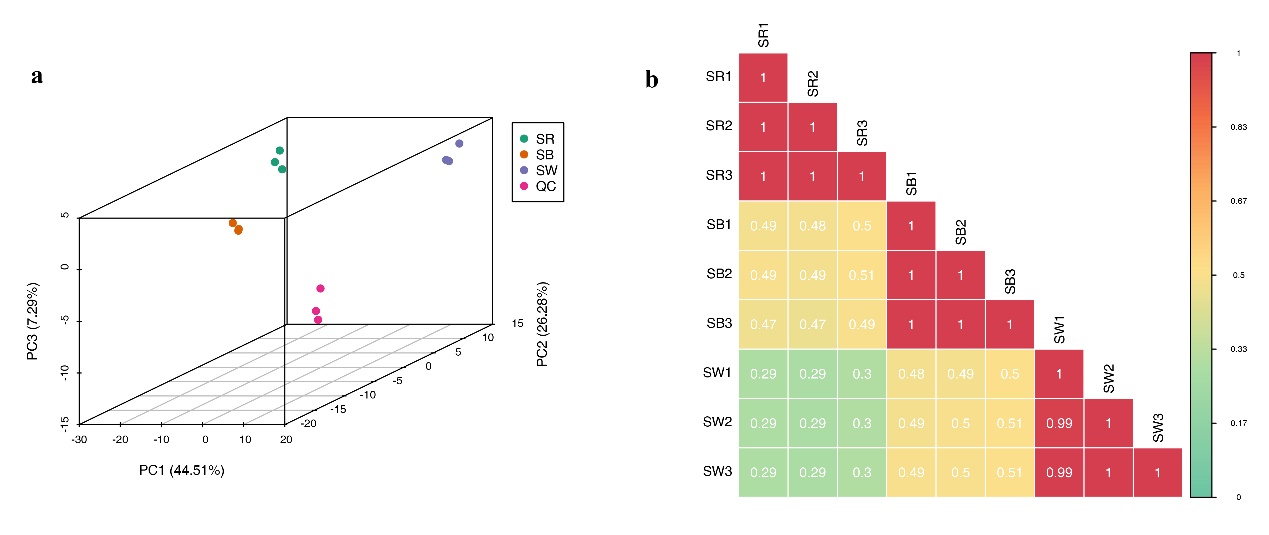


**Additional file 2: Figure S1.** Principal component analysis and repeated correlation assessment of metabolome data

a. Principal component analysis among samples. PC1 represents the first principal component, PC2 represents the second principal component, PC3 represents the third principal component, and percentage represents the interpretation rate of this principal component to the data set. Each point in the figure represents a sample, and samples in the same Group are represented by the same color. Different group are distinguished by different colours. b. Correlation analysis among samples. The vertical and diagonal lines represent different sample names, and different colours represent different Pearson correlation coefficients.
