# Supplementary material for: Integrated metabolome and transcriptome analyses of anthocyanin biosynthesis reveal key candidate genes involved in colour variation of Scutellaria baicalensis flowers
Source: BMC Plant Biol. 2023 Dec 15;23:643. doi: 10.1186/s12870-023-04591-3 (PMC10722828; doi:10.1186/s12870-023-04591-3)
Supplement: Supplementary file 3 — Additional file 3: Figure S2. The top 20 enriched KEGG pathways of DAMs shown by DA score in SB vs SR (a), SB vs SW (b) and SW vs SR (c). X axis represents the DA score. Y axis represents KEGG pathways. DA Score reflects the overall change of metabolites. A score of 1 indicates an upward trend in the expression of all identified metabolites in this pathway, and a score of -1 indicates a downward trend. The length of the line segment represents the absolute value of the DA Score. The dot size indicates the number of differentiated metabolites in the pathway, and the larger the dot, the more metabolites. The color of the line segment and dot reflects the P-value size. The closer it is to red, the smaller the P-value, and the closer it is to purple, the larger the P-value. [file 12870_2023_4591_MOESM3_ESM.docx]

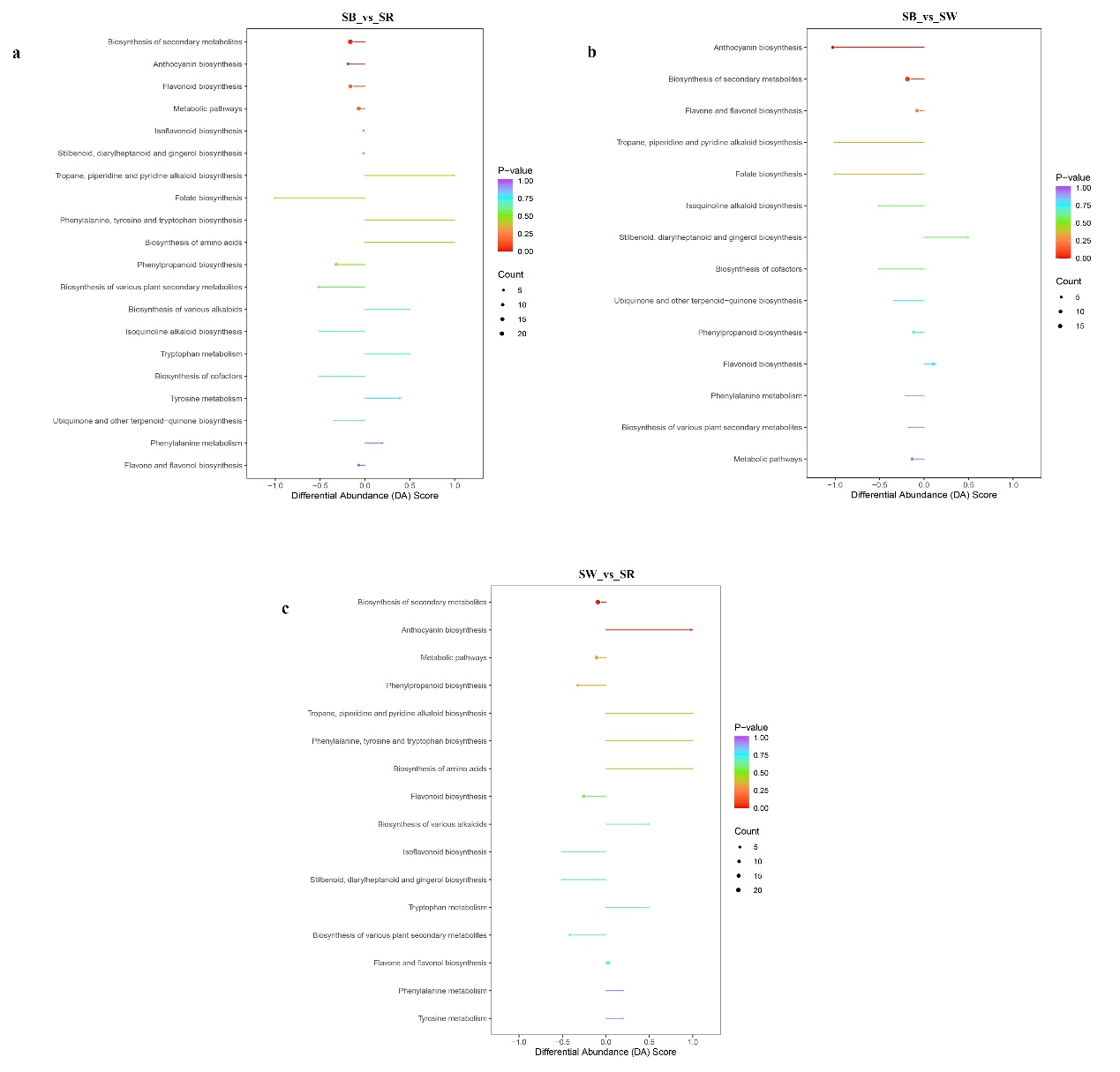


**Additional file 3: Figure S2.** The top 20 enriched KEGG pathways of DAMs shown by DA score in SB vs SR (a), SB vs SW (b) and SW vs SR (c)

X axis represents the DA score. Y axis represents KEGG pathways. DA Score reflects the overall change of metabolites. A score of 1 indicates an upward trend in the expression of all identified metabolites in this pathway, and a score of -1 indicates a downward trend. The length of the line segment represents the absolute value of the DA Score. The dot size indicates the number of differentiated metabolites in the pathway, and the larger the dot, the more metabolites. The color of the line segment and dot reflects the P-value size. The closer it is to red, the smaller the P-value, and the closer it is to purple, the larger the P-value.
