# Supplementary material for: Integrated metabolome and transcriptome analyses of anthocyanin biosynthesis reveal key candidate genes involved in colour variation of Scutellaria baicalensis flowers
Source: BMC Plant Biol. 2023 Dec 15;23:643. doi: 10.1186/s12870-023-04591-3 (PMC10722828; doi:10.1186/s12870-023-04591-3)
Supplement: Supplementary file 4 — Additional file 4: Figure S3. Principal component analysis and correlation analysis of transcriptome data. a. Principal component analysis among samples. b. Correlation analysis among samples. [file 12870_2023_4591_MOESM4_ESM.docx]

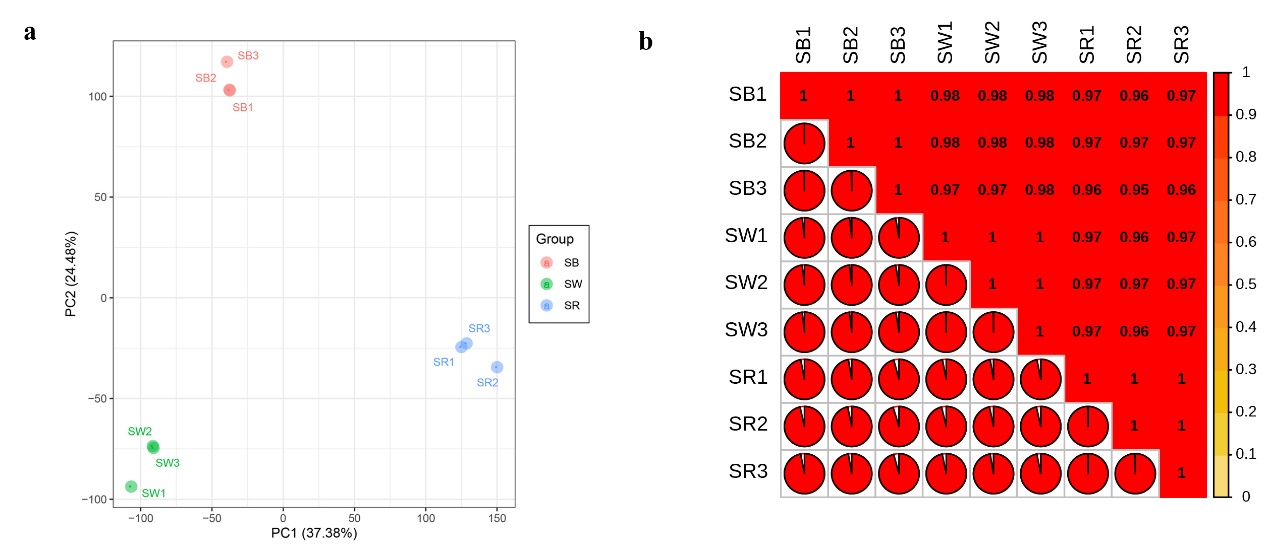


**Additional file 4: Figure S3.** Principal component analysis and correlation analysis of transcriptome data

a. Principal component analysis among samples. b. Correlation analysis among samples.
