# Supplementary material for: Integrated metabolome and transcriptome analyses of anthocyanin biosynthesis reveal key candidate genes involved in colour variation of Scutellaria baicalensis flowers
Source: BMC Plant Biol. 2023 Dec 15;23:643. doi: 10.1186/s12870-023-04591-3 (PMC10722828; doi:10.1186/s12870-023-04591-3)
Supplement: Supplementary file 10 — Additional file 10: Figure S4. Expression patterns of structural genes and main TFs associated with anthocyanin biosynthesis in different tissues of S. baicalensis. The gene expression level was expressed by TPM value and normalized by row. The redder cells indicate higher expression, and greener cells indicate lower expression. [file 12870_2023_4591_MOESM10_ESM.docx]

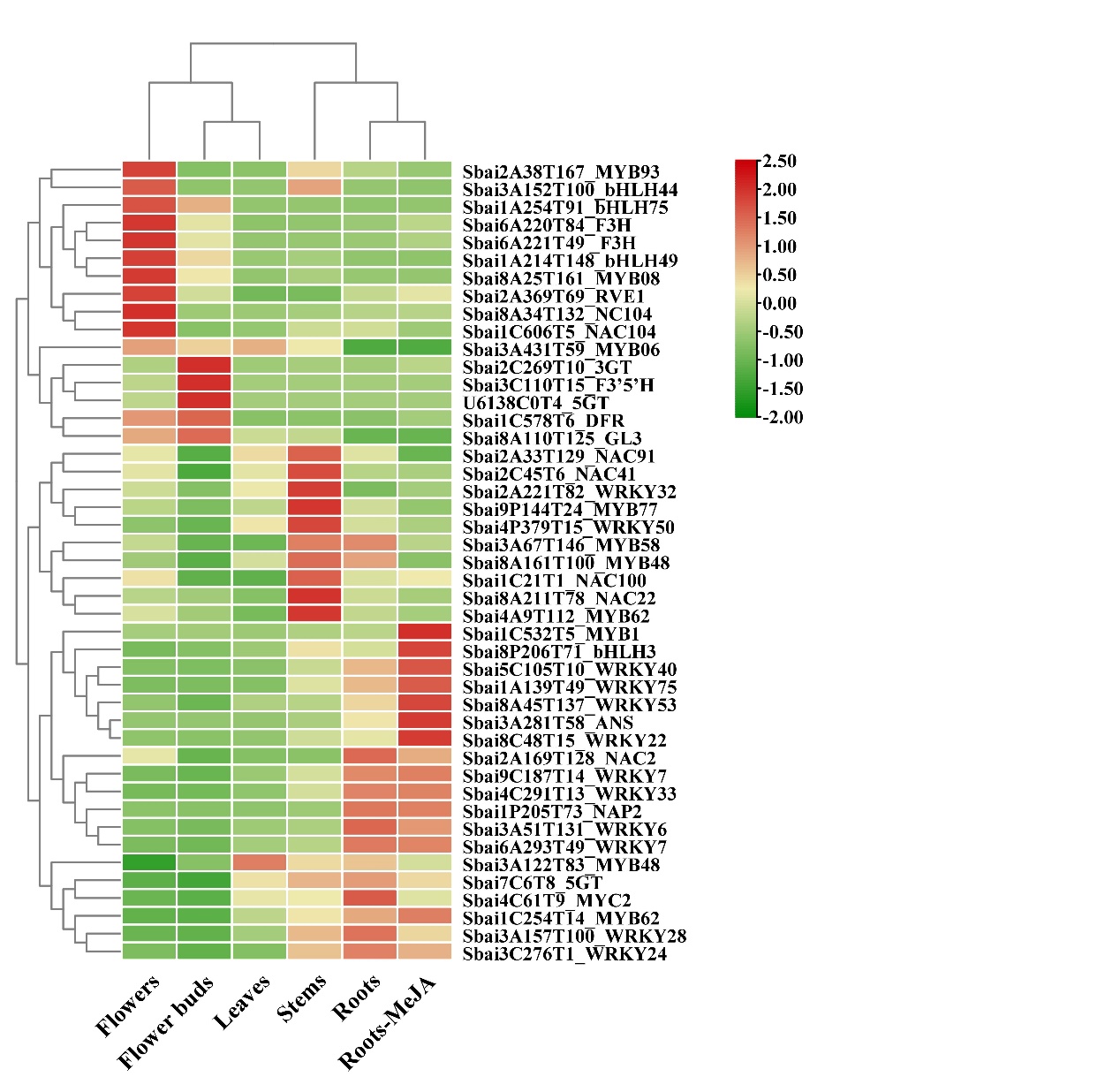


**Additional file 10: Figure S4.** Expression patterns of structural genes and main TFs associated with anthocyanin biosynthesis in different tissues of *S. baicalensis*

The gene expression level was expressed by TPM value and normalized by row. The redder cells indicate higher expression, and greener cells indicate lower expression.
